# Analysing and reporting of observational data: a systematic review informing the EULAR points to consider when analysing and reporting comparative effectiveness research with observational data in rheumatology

**DOI:** 10.1136/rmdopen-2021-001818

**Published:** 2021-11-17

**Authors:** Kim Lauper, Joanna Kedra, Maarten de Wit, Bruno Fautrel, Thomas Frisell, Kimme L Hyrich, Florenzo Iannone, Pedro M Machado, Lykke M Ørnbjerg, Ziga Rotar, Maria Jose Santos, Tanja A Stamm, Simon R Stones, Anja Strangfeld, Robert BM Landewé, Axel Finckh, Sytske Anne Bergstra, Delphine S Courvoisier

**Affiliations:** 1 Division of Rheumatology, Geneva University Hospitals & Faculty of Medicine, University of Geneva, Geneva, Switzerland; 2 Centre for Epidemiology Versus Arthritis, Manchester Academic Health Sciences Centre, The University of Manchester, Manchester, UK; 3 Institut Pierre Louis d'Epidémiologie et de Santé Publique (iPLESP), UMR S 1136, Sorbonne Universite, Paris, France; 4 APHP, Rheumatology Department, Pitié Salpêtrière Hospital, Paris, France; 5 Patient Research Partner, EULAR, Zaltbommel, The Netherlands; 6 Clinical Epidemiology Division, Department of Medicine (Solna), Karolinska Institutet, Stockholm, Sweden; 7 NIHR Manchester Biomedical Research Centre, Manchester University Hospitals NHS Foundation Trust, Manchester, UK; 8 8Department of Emergency and Organ Transplantation, Rheumatology Unit, University of of Bari, Bari, Italy; 9 Centre for Rheumatology & Department of Neuromuscular Diseases, University College London, London, UK; 10 National Institute for Health Research (NIHR) Biomedical Research Centre, University College London Hospitals NHS Foundation Trust, London, UK; 11 Copenhagen Center for Arthritis Research, Center for Rheumatology and Spine Diseases, Centre for Head and Orthopedics, Rigshospitalet, Glostrup, Denmark; 12 DANBIO registry, Center for Rheumatology and Spine Diseases, Centre for Head and Orthopedics, Rigshospitalet, Copenhagen, Denmark; 13 BioRx.si, Department of Rheumatology, University Medical Centre Ljubljana & Faculty of Medicine, University of Ljubljana, Ljubljana, Slovenia; 14 Reuma.pt registry, Rheumatology Research Unit, Instituto de Medicina Molecular, Faculdade Medicina de Lisboa, Lisboa, Portugal; 15 Rheumatology Department, Hospital Garcia de Orta, Almada, Portugal; 16 Section for Outcomes Research, Centre for Medical Statistics, Informatics and Complex Systems, Medical University of Vienna, Wien, Austria; 17 EULAR patient research partner, Manchester, UK; 18 Programme area Epidemiology, Deutsches Rheuma-Forschungszentrum (DRFZ), Berlin, Germany; 19 Clinical Immunology & Rheumatology, Academic Medical Center, University of Amsterdam, Amsterdam, The Netherlands; 20 Department of Rheumatology, Zuyderland Medical Center, Heerlen, The Netherlands; 21 Department of Rheumatology, Leiden University Medical Center, Leiden, The Netherlands

**Keywords:** epidemiology, antirheumatic agents, arthritis

## Abstract

**Objectives:**

To evaluate the analysis and reporting of comparative effectiveness research with observational data in rheumatology, informing European Alliance of Associations for Rheumatology points to consider.

**Methods:**

We performed a systematic literature review searching Ovid MEDLINE for original articles comparing drug effectiveness in longitudinal observational studies, published in key rheumatology journals between 2008 and 2019. The extracted information focused on reporting and types of analyses. We evaluated if year of publication impacted results.

**Results:**

From 9969 abstracts reviewed, 211 articles fulfilled the inclusion criteria. Ten per cent of studies did not adjust for confounding factors. Some studies did not explain how they chose covariates for adjustment (9%), used bivariate screening (21%) and/or stepwise selection procedures (18%). Only 33% studies reported the number of patients lost to follow-up and 25% acknowledged attrition (drop-out or treatment cessation). To account for attrition, studies used non-responder imputation, followed by last observation carried forward (LOCF) and complete case (CC) analyses. Most studies did not report the number of missing data on covariates (83%), and when addressed, 49% used CC and 11% LOCF. Date of publication did not influence the results.

**Conclusion:**

Most studies did not acknowledge missing data and attrition, and a tenth did not adjust for any confounding factors. When attempting to account for them, several studies used methods which potentially increase bias (LOCF, CC analysis, bivariate screening…). This study shows that there is no improvement over the last decade, highlighting the need for recommendations for the assessment and reporting of comparative drug effectiveness in observational data in rheumatology.

**Key messages**

**What is already known about this subject?**

- While the quality of observational studies in medicine has already been studied, little is known about the quality of observational research in rheumatology, especially in the field of comparative effectiveness.

**What does this study add?**

- This study demonstrates that the analysis and the reporting of comparative effectiveness studies in rheumatology needs to be improved, in particular the management of confounding, attrition and missing data.

**How might this impact on clinical practice or further developments?**

- More robust comparative effectiveness research may help to support everyday clinical decisions with high-quality evidence.

## Introduction

Observational data are increasingly used in rheumatology for safety and effectiveness analyses of new therapies, and are progressively more required by health authorities in regulatory processes.^1^ In randomised controlled trials (RCTs), comparing the efficacy across drugs is relatively straightforward since treatment groups should be similar in terms of patient characteristics by means of an adequate randomisation process. However, with their strict inclusion criteria, short follow-up and placebo comparators, RCTs are not always helpful for clinical decision making. Observational studies are thus invaluable for their insights into ‘real-life’, and can be used to assess effectiveness (ie, how well a treatment performs in routine clinical settings). However, observational studies have a higher potential for bias and confounding.^2 3^ Missing data are more often an issue and differences in the follow-up (drop-out) in the treatment groups can also lead to selection bias when analysing the outcome (attrition bias).^3^

The European Alliance of Associations for Rheumatology (EULAR) has previously published recommendations on the analysis and reporting of observational safety data in biologic drug registers and clinical trial extension studies.^4 5^ International initiatives, such as STrengthening the Reporting of OBservational studies in Epidemiology (STROBE), provide guidance on what should be reported in observational studies.^6^ However, there are no detailed recommendations on how comparative effectiveness research (CER) studies should be adequately analysed and reported in rheumatology. This systematic literature review (SLR) aims to evaluate how CER studies are currently analysed and reported in this field to determine the quality and the variability of the information reported and the analytical procedures used. These findings underpin the development of EULAR points to consider for the analysis and reporting of CER with observational data.

## Methods

The results of this SLR were reported according to the Preferred Reporting Items for Systematic Reviews and Meta-Analyses guidelines,^4^ when applicable. The EULAR standardised operating procedures were also followed.^7^ The search strategy was formulated and reviewed during the first multistakeholder EULAR Task Force meeting, including patient research partners, healthcare professionals and researchers. The preliminary findings were subsequently presented and discussed during the second EULAR Task Force meeting. The Task force comprised a multidisciplinary team of experts including rheumatologists, health professionals, patients, statisticians and epidemiologists.

### Search strategy

Since the aim of the SLR was not to conduct a meta-analysis, but to qualitatively assess CER in rheumatology, focusing on reporting and analyses, we restricted the target journals to leading rheumatology journals indexed in one database (Ovid MEDLINE). In order to select higher quality articles and to evaluate contemporary research, the search was limited to journals with a Scientific Journal Ranking (SJR)^5^ of two or more as of 25 March 2019 and to publications in the last 10 years (1 January 2008 to 25 March 2019). In addition, because no standard keywords exist to indicate CER studies, exclusion keywords only were used to preclude RCTs, genetic studies and so on (online supplemental table 1).

10.1136/rmdopen-2021-001818.supp1 Supplementary data

### Study selection

Studies were included if they were original longitudinal observational CER studies with ≥100 participants and exploring effectiveness of different drugs, even if treatment was not the main exposure, nor effectiveness the only outcome. We decided a priori not to include smaller studies (<100 participants), because they could have additional methodological issues that were felt to be out of the scope for this Task Force. Furthermore, studies evaluating extra-articular organ damage were not included as it was felt that this would be a different outcome and thus may need different type of analyses or reporting, as reversibility is rarely achievable.

Considering the numbers of articles to screen and the aim of the SLR to qualitatively assess CER, the first 1000 titles and abstracts were screened independently by two reviewers (KL and DC) to identify potentially eligible studies. Inter-rater reliability was then evaluated using percentage of agreement and Cohen’s kappa. Subsequent abstracts were screened by only one reviewer (KL or DC) as agreement was deemed sufficient (agreement with a kappa of 0.6) by the Taskforce at the initial meeting. Potentially eligible studies were reviewed in full text, and those fulfilling the inclusion criteria were then proceeded to data extraction.

### Data extraction

Data extraction was performed systematically using a standardised form (online supplemental table 2) by KL and DC based on the items discussed during the first meeting of the Task Force. The following data were extracted from the studies: general information about the study, study methods, definition of exposures and outcomes, and handling of confounding, attrition and missing data (see online supplemental table 2 for full list and online supplemental table 3 for definitions).

### Analyses

**Figure 1:**
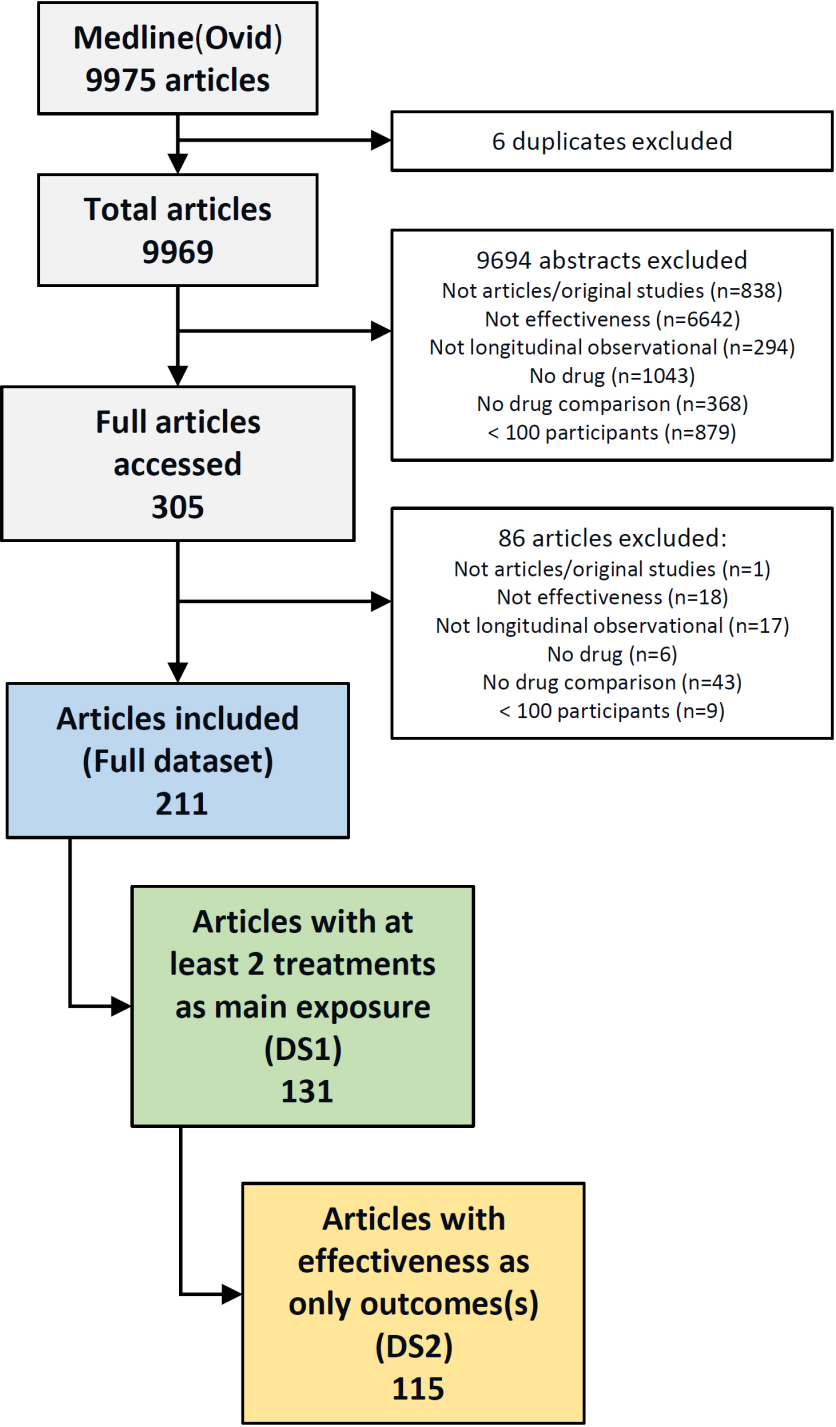
Diagram depicting the screening process of studies included in the systematic literature review.

Standard descriptive statistics were used for the analyses. Two sensitivity analyses were performed, to explore if the quality of analysis and reporting of CER impacted the findings of this SLR. To see if studies focusing only on CER (eg, treatment comparison and effectiveness) improved reporting and analyses, we created two subsets of data. In the first dataset (DS1), we included only studies where the comparison of at least two treatments was the main exposure of interest (head-to-head studies, figure 1). In the second dataset (DS2), we included only studies from DS1 focusing exclusively on effectiveness, as we wanted to assess if studies with only effectiveness outcomes, with more space available in the manuscript and more time to dedicate to this outcome, could have had better reporting and analyses. We further evaluated if the reporting and analyses varied by year of publication or by SJR, from the lowest ranking (category 1) to the highest (category 8).

## Results

The initial literature search yielded 9975 references (9969 unique). Figure 1 describes the selection processes. From the 9969 abstracts screened, 305 full-text articles were assessed for eligibility; with 211 articles proceeded to data extraction (figure 1, online supplemental file 3). The main reasons for exclusion of original studies were the absence of at least one effectiveness outcome, of treatment comparison and small cohorts (<100 participants).

10.1136/rmdopen-2021-001818.supp3 Supplementary data

### General information on the studies

A range of different rheumatic and musculoskeletal diseases were included, with the most frequent being rheumatoid arthritis (117 studies), followed by spondyloarthritis (52 studies) and juvenile idiopathic arthritis (16 studies).

### General information on methods

All articles had a method section (table 1). Data collection was prospective in most studies (174 studies, 82%). For 18 studies (9%), there was no mention of how the data were collected. Ten studies (5%) explicitly stated that they followed the STROBE guidelines when reporting their study findings.^6^

**Table 1:** Summary of results for reporting and analysing for general information and outcomes

	Main analysis	DS1	DS2
**N**	211	131	115
General information on methods			
Description of the data collection			
Prospective	174 (82%)	106 (81%)	94 (82%)
Retrospective	18 (9%)	13 (10%)	11 (10%)
Mixed	1 (0%)	1 (1%)	1 (1%)
Not mentioned	18 (9%)	10 (8%)	9 (8%)
Indication of adherence to STROBE reporting guidelines	10 (5%)	6 (5%)	6 (5%)
Outcomes			
More than one effectiveness outcome	107 (51%)	78 (60%)	69 (60%)

DS1: Dataset 1 includes only studies where the comparison of at least two treatments was the main exposure of interest (head to head studies).

DS2: Dataset 2 includes only studies from DS1, which had no other outcome than effectiveness.

STROBE, STrengthening the Reporting of OBservational studies in Epidemiology.

### Exposures and outcomes

In 53 studies (25%), treatment was not the main exposure, but evaluated secondarily in the study. Thirty-eight studies (18%) had at least one outcome beside effectiveness (mostly safety). For 131 studies (62%), treatment was the main exposure, with comparisons of at least 2 treatments and comprised the first dataset (DS1, figure 1); of these, 115 studies (55%) focused on treatment effectiveness outcomes (DS2).

A total of 107 studies (51%) reported more than one type of effectiveness outcome (table 1); though this proportion was higher when restricting to DS1 (60%) or DS2 (60%). No clear trend in the percent of studies reporting only a single effectiveness outcome over time or by SJR were found (online supplemental figure 1). The most frequent effectiveness outcome was disease activity, reported as a categorical outcome, followed by drug retention.

### Confounding

**Figure 2:**
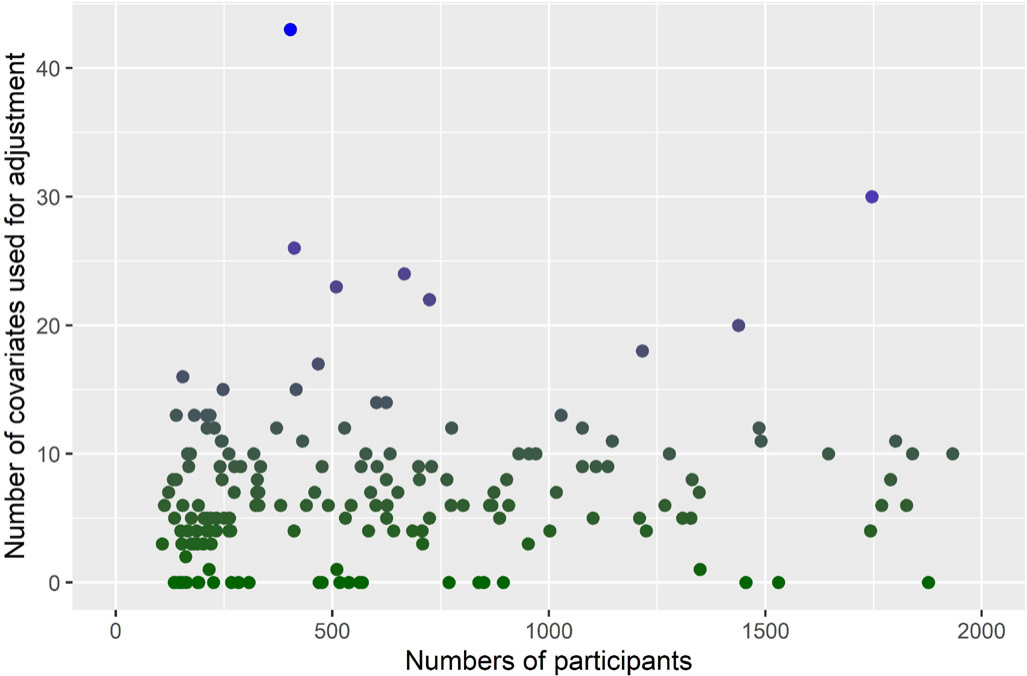
Number of covariates used for adjustment by number of participants for studies including between 100 and 2000 participants.

Thirty studies (14%) did not present a crude and adjusted analysis (table 2), with 22 (10%) studies presenting only a crude analysis and 8 (4%) only an adjusted analysis. The results were similar in the analyses by publication year and SJR, in DS1 and DS2 and between studies mentioning STROBE or not (online supplemental table 4). The most common method to select variables for adjustment was a priori selection of covariates (114 studies, 54%). Twenty studies (9%) did not explain what method was used to select covariates, 45 (21%) used univariate/bivariate screening, 37 (18%) used a data-driven stepwise approach (eg, forward selection and backward elimination …) (online supplemental figure 2, online supplemental table 3). No studies reported using more advanced variable selection methods, such as least absolute shrinkage and selection operator or Elastic Net methods. We found no clear trend by year of publications (online supplemental figure 2A) or SJR (online supplemental figure 2B). For the number of covariates used for adjustment, three studies (1%) did not mention how many covariates or which covariates were used. We found no correlation between the number of covariates used for adjustment and the number of participants, with some very small studies using a high number of covariates and some large studies using none (figure 2). The most common method used for adjustment was a multivariable model (146 studies, 69%).

**Table 2:** Summary of results for reporting and analysing for confounding

	Main analysis	DS1	DS2
**N**	211	131	115
Adjusted and crude analysis presented	181 (86%)	112 (85%)	100 (87%)
Crude analysis presented only	22 (10%)	12 (9%)	9 (8%)
Adjusted analysis presented only	8 (4%)	8 (6%)	6 (5%)
Method of selection for adjustment covariates*			
A priori/wisely	114 (54%)	77 (59%)	69 (60%)
Stepwise method	37 (17%)	19 (15%)	15 (13%)
Bivariate selection	45 (21%)	25 (19%)	21 (18%)
Unknown	20 (9%)	12 (9%)	11 (10%)
Other	1 (0%)	1 (1%)	1 (1%)
Method of adjustment for confounding*			
Multivariable model	146 (69%)	88 (67%)	88 (77%)
Stratification	9 (4%)	5 (4%)	5 (4%)
Matching	17 (8%)	14 (11%)	14 (12%)
Inverse probability weighting	7 (3%)	5 (4%)	5 (4%)
Propensity score	26 (12%)	23 (18%)	23 (20%)
Restriction	0	0	0
Other	1 (0%)	0	0

DS1: Dataset 1 includes only studies where the comparison of at least two treatments was the main exposure of interest (head to head studies).

DS2: Dataset 2 includes only studies from DS1, which had no other outcome than effectiveness.

*Sum of the methods may be greater than the numbers of studies as some studies used several methods.

### Follow-up and attrition

**Figure 3:**
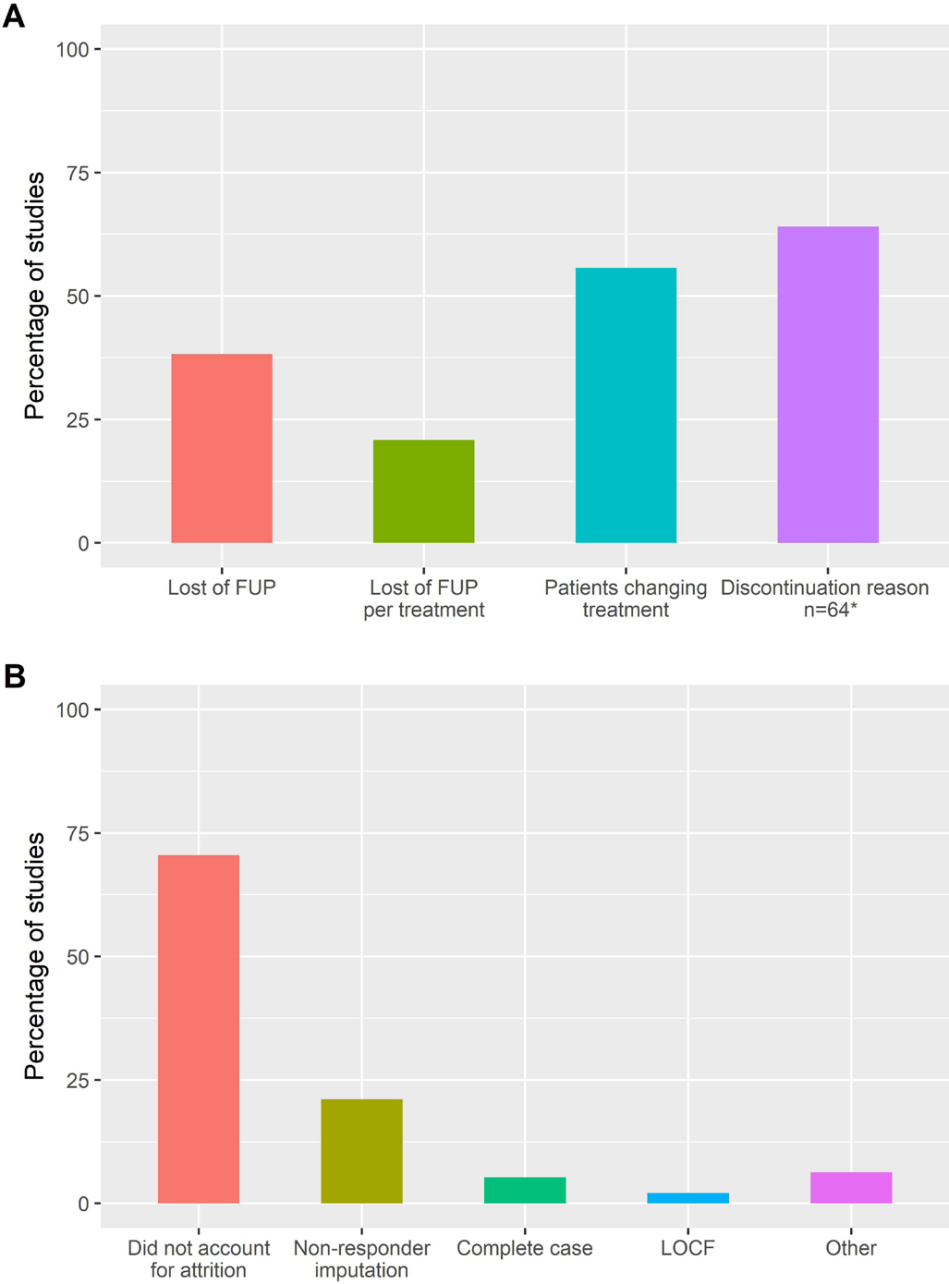
Percentage of studies (A) reporting of follow-up information and (B) using a certain method to handle attrition, in studies with at least two treatments as the main exposure and only effectiveness as the outcomes (DS2, n=115). *For discontinuation reason, the denominator are studies that reported the patients changing treatment (n=64). FUP, follow-up; LOCF, last observation carried forward.

Sixty-nine studies (33%) presented the overall number of patients lost to follow-up (no longer under the treatment of interest or in the cohort for any reasons such as death, loss of follow-up, migration, change of treatment, …)(table 3). The percentages were in the same range in our sensitivity analyses for DS1 (36%) and DS2 (38%). Thirty studies (14%) further presented the number of lost to follow-up by treatment in the main analysis, with a slightly higher proportion for DS1 (20%) and DS2 (21%) (figure 3A). A total of 101 studies (48%) reported the number of patients who stopped or changed treatment. The percentage slightly increased in studies where at least two treatments were the main exposures, with 57% in DS1 and 56% in DS2 (figure 3A).

**Table 3:** Summary of results for reporting and analysing for follow-up information and handling of attrition

	Main analysis	DS1	DS2
**N**	211	131	115
Reporting of patients lost to follow-up	69 (33%)	47 (36%)	44 (38%)
Reporting of patients lost to follow-up by treatment	30 (14%)	26 (20%)	24 (21%)
Reporting of patients changing/stopping treatment	101 (48%)	75 (57%)	64 (56%)
Reporting of reasons for treatment discontinuation	62 (29%)	47 (36%)	41 (36%)
Handling of attrition in the analysis (in studies with outcome other than retention)	44/177 (25%)	31/106 (29%)	28/95 (29%)
Method to handle attrition (when acknowledged)*			
Non-responder imputation	27/44 (62%)	22/31 (71%)	20/28 (72%)
Complete case	8/44 (18%)	5/31 (16%)	5/28 (18%)
Last observation carried forward	8/44 (18%)	4/31 (12%)	2/28 (7%)
Other	8/44 (18%)	7/31 (23%)	6/28 (21%)

DS1: Dataset 1 includes only studies where the comparison of at least two treatments was the main exposure of interest (head to head studies).

DS2: Dataset 2 includes only studies from DS1, which had no other outcome than effectiveness.

*Sum of the methods may be greater than the numbers of studies as some studies used several methods.

For the reasons of drug discontinuation, looking only at studies that reported the number of patients that stopped or changed treatment, 39% of the studies examining this outcome did not report the reasons for discontinuation for those patients. A similar proportion was found in our sensitivity analyses in DS1 and DS2 (37% and 36% not mentioning the discontinuation reasons, respectively). For studies evaluating at least another outcome than retention (177 studies), 133 (75%) did not take into account attrition in the analysis. Similar proportions were found in sensitivity analyses of studies focusing on CER (71% in DS1 and 71% in DS2, figure 3B). Among the 44 studies that accounted for attrition, the most frequent method used to adjust for attrition was a non-responder imputation, categorising patients no longer under the treatment of interest as non-responders (27 studies, 62%). Eight studies (18%) used ‘complete-case analyses’, and eight studies (18%) used’ last observation carried forward’ (LOCF) methods to impute the outcome.

There were no major differences in reporting or analyses for studies mentioning STROBE or not for main categories (online supplemental table 1).

### Missing data on covariates of interest

**Figure 4:**
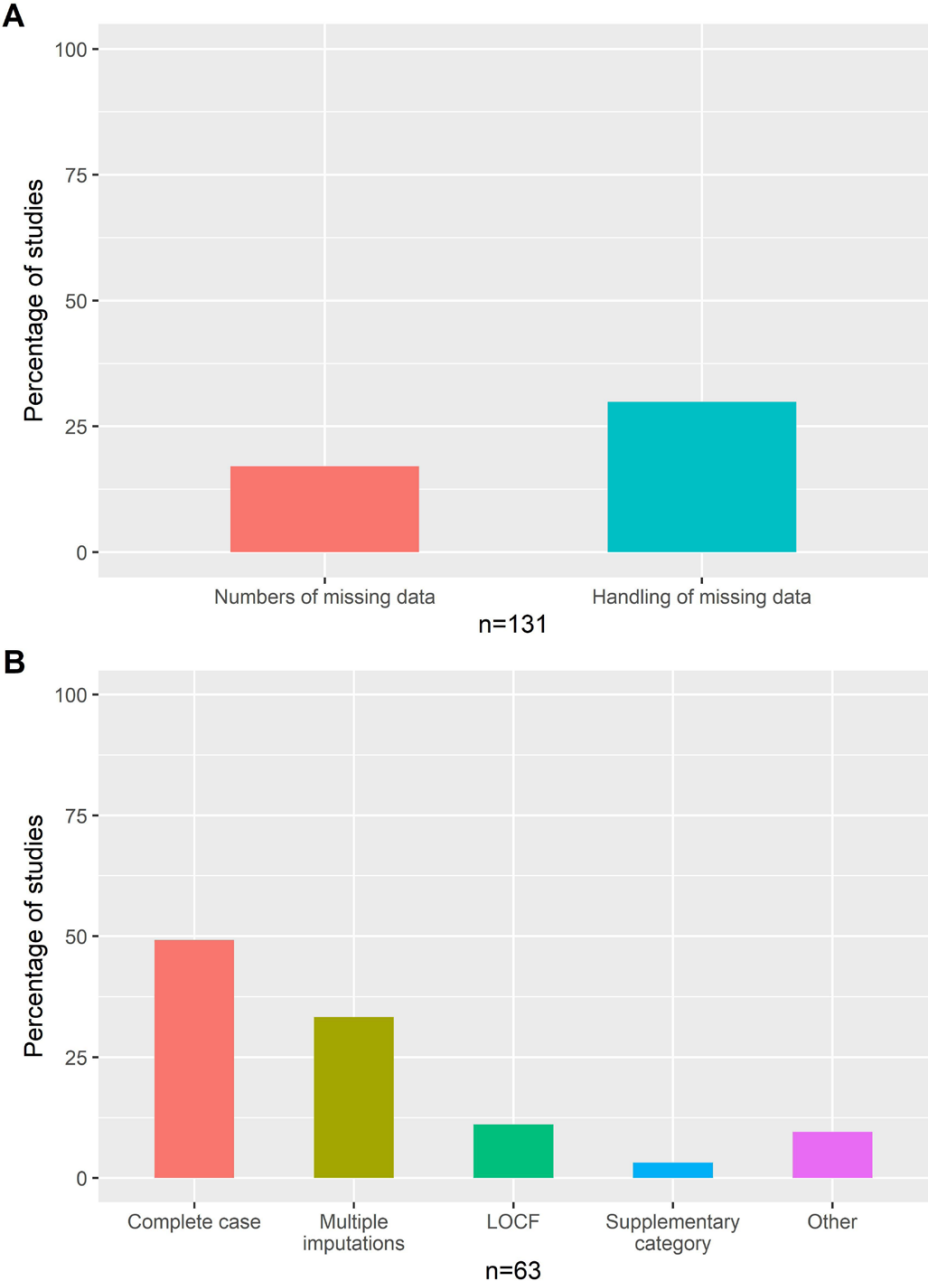
Percentage of studies (A) reporting information on missing data and handling of missing data (in all studies, n=131) and (B) by method of handling missing data (for studies that reported how they handled missing data, n=63). LOCF, last observation carried forward.

The majority of studies (83%) did not report the number of missing data for covariates of interest and 148 studies (70%) did not report how missing data were handled (figure 4A, table 4). In the 63 studies that reported on missing data handling, complete-case analysis was the most frequent technique used (31 studies, 49%), followed by multiple imputation methods (21 studies, 33%), and LOCF for at least one covariate (7 studies, 11%) (figure 4B). There was no trend by publication year or SJR ranking (online supplemental figure 4). Studies mentioning STROBE did not seem to report better the number of missing data or the methods to handle them (online supplemental table 1).

**Table 4:** Summary of results for reporting and analysing for missing data

	Main analysis	DS1	DS2
**N**	211	131	115
Reporting of the no of missing data of covariates of interest	36 (17%)	22 (17%)	20 (17%)
Reporting of the method to handle missing data	63 (30%)	39 (30%)	36 (31%)
Handling of missing data*			
Complete case	31 (15%)	18 (14%)	16 (14%)
Multiple imputation	21 (10%)	13 (9%)	12 (10%)
Last observation carried forward	7 (3%)	4 (3%)	4 (3%)
Supplementary category	2 (1%)	2 (2%)	2 (2%)
Other	5 (3%)	3 (2%)	3 (3%)

DS1: Dataset 1 includes only studies where the comparison of at least two treatments was the main exposure of interest (head to head studies).

DS2: Dataset 2 includes only studies from DS1, which had no other outcome than effectiveness.

*Sum of the methods may be greater than the numbers of studies as some studies used several methods.

## Discussion

Our SLR demonstrates that currently, many CER studies in rheumatology inadequately report on covariates of interest for adjustment, follow-up information, treatment changes and missing data, which hinders the interpretation of such studies. In addition, some of the methods used to mitigate these issues are debatable. These trends have not changed over the past 10 years or by SJR. This is worrying as inadequate methods and reporting contributes to the waste of valuable resources, as articles may not be usable.^6 7^

Compared with RCTs, comparison groups in observational cohorts may not be similar, and some patients’ characteristics may be associated with treatment assignment. For rheumatoid arthritis, for example, patients treated with tocilizumab tend to be older and have longer disease durations than patients treated with tumour necrosis factor-inhibitors.^8^ If these characteristics are also associated with the outcome, they may confound the association between the exposure and the outcome, thus introducing bias. In theory, confounding could be accounted for by statistical adjustments methods. However, this was seldom performed. In addition, there was no clear correlation between the number of participants (as a proxy for the number of events) and the number of covariates used for adjustment, with some small studies using a high number of covariates, which could lead to incorrect estimation of parameters or overadjustment.^9^ On the other hand, some studies with large number of participants did not adjust at all for confounding. When selecting confounders, the most frequent method used was bivariate screening, which may overlook important confounding factors and produce incorrect estimates.^10^ Other studies used stepwise approaches (eg, backward elimination, and forward selection), which are not designed to select confounders but instead covariates which are associated with the outcome.

Attrition is a type of selection bias that can occur if participants are lost to follow-up or cannot contribute a value of effectiveness. This bias can be differential by exposure if attrition differ by treatment. The results can vary due to the reasons for loss of follow-up. In a review of RCTs, 19% of trials lost their significance when loss to follow-up participants were assumed not to have the event of interest.^11^ In a simulation study, differential attrition greatly biased the association between the covariates of interest and the outcome.^8^ In the minority of studies that considered attrition, the main method was a non-responder imputation, which is a very conservative approach: it assumes that all patient lost to follow-up did not respond to therapy. Since patients can discontinue their treatments for various reasons (eg, adverse events, pregnancy and remission), this assumption is clearly incorrect in some cases. Other studies used LOCF, which is unbiased only under specific situations (data missing completely at random, ie, independently of any other variables),^12^ or complete-case analysis, which can lead to loss of power and biased results if values are not missing completely at random.^13^ The same findings also apply to missing covariates, where complete-case analysis was the most commonly reported method for studies describing how missing data were handled, followed by multiple imputation and LOCF. Multiple imputations may be a better choice when data are missing at random (online supplemental table 3).^14^

Most of the CER studies included only explored one aspect of effectiveness (one outcome). Considering the many possibilities of biases and confounding, using only one type of outcome to answer a research question may not be enough to shape a comprehensive picture. Additionally, distinct treatment may influence various aspects of the disease differently.

This study is consistent with previous SLRs of methods of CER in rheumatology, which have demonstrated that study designs and reporting could be improved. In a study including 78 rheumatology papers, one in six did not account for time-dependent biases.^15^ Another review on 35 CER studies of rheumatoid arthritis, exploring a different aspect of the quality of studies than in our current review, found that 61% used postbaseline information to evaluate eligibility at baseline.^16^ In this study, the authors also mention the lack of adjustment for confounding factors and the use of statistical methods only to select the variables included in the model (bivariate screening, stepwise approach), as in our SLR. Systematic reviews in other fields have shown that this finding is however not pertained to rheumatology research.^17 18^ Information on lost to follow-up and its handling were often unclear in a systematic review of RCTs in internal medicine journals.^11^

Several tools exist to improve the quality of reporting of observational studies such as STROBE,^3^ which ask to report how missing data are handled, the number of missing data for each covariates of interest and follow-up time information, among other information. Some academic societies, such as the EULAR, also have developed support for researcher aiming to improve research quality at every steps, including for analyses.^9^

One of the limitations of this study is that we did not include all rheumatology articles, from every journal, and used only one indexed database. However, the aim of this SLR was to evaluate the main issues concerning CER in current rheumatology literature, an exercise that unlike conventional SLRs, did not require a larger pool of journals. In addition, our SLR covered the main journals in rheumatology, and most likely also those publishing the highest quality CER in the field. Use of SJR as a selection criterion for studies could also be arguable; however, we wanted to be able to include some recent journals that may not currently have an impact factor, as the aim was to evaluate contemporary research. Importantly, it is also possible that some information was not included in the manuscript because of the word limitations, which we did not evaluate. However, online supplemental materials, which are not dependent on word limits, were also screened. We found a low number of studies explicitly mentioning that they complied with STROBE recommendations. For feasibility reasons, we did not evaluate every item from the STROBE checklist. It is thus possible that more studies were reporting according to STROBE as journals, without mentioning it in the manuscript or adding it as online supplemental materials. The number of studies mentioning to report according to STROBE was very low (10 studies), so any potential differences with studies not mentioning STROBE may be due to chance only. The strength of this study is that we reviewed key aspects of CER and included a broad selection of published observational research papers.

In conclusion, this SLR confirms that reporting and analysis of observational CER needs to be improved in rheumatology, particularly on aspects of confounding, missing data on the covariates and attrition. Because this study shows there is no improvement over the last decade there is a need for additional recommendations for the assessment and reporting of comparative drug effectiveness in observational data in rheumatology. This SLR has been used to inform the ‘EULAR points to consider when analysing and reporting CER with observational data in rheumatology’, which will help to set standards to help improve the quality of CER in rheumatology.

10.1136/rmdopen-2021-001818.supp2 Supplementary data

## Data Availability

Data are available as supplementary materials (please see online supplementary file 2). More information on the data are available on request from the authors. Not applicable.
