# Supplementary material for: Analysing and reporting of observational data: a systematic review informing the EULAR points to consider when analysing and reporting comparative effectiveness research with observational data in rheumatology
Source: RMD Open. 2021 Nov 17;7(3):e001818. doi: 10.1136/rmdopen-2021-001818 (PMC8601074; doi:10.1136/rmdopen-2021-001818)

SUPPLEMENTARY MATERIAL

Supplementary Table 1: Search Strategy on Ovid MEDLINE®

1. "annals of the rheumatic diseases".jn.
2. arthritis & rheumatology.jn.
3. arthritis & rheumatism.jn.
4. arthritis care & research.jn.
5. seminars in arthritis & rheumatism.jn.
6. rheumatology.jn.
7. rmd open.jn.
8. "journal of rheumatology".jn.
9. arthritis research & therapy.jn.
10. 1 or 2 or 3 or 4 or 5 or 6 or 7 or 8 or 9
11. exp Journal Article/
12. 10 and 11
13. ((("semi-structured" or semistructured or unstructured or informal or "in-depth" or indepth or "face-to-face" or structured or guide) adj3 (interview* or discussion*)) or (focus group* or qualitative or ethnograph* or fieldwork or "field work" or "key informant")).ti,ab. or interviews as topic/ or focus groups/ or narration/ or qualitative research/
14. Meta-Analysis as Topic/ or meta analy$.tw. or metaanaly$.tw. or Meta-Analysis/ or (systematic adj (review$1 or overview$1)).tw. or exp Review Literature as Topic/ or cochrane.ab. or embase.ab. or (psychlit or psyclit).ab. or (psychinfo or psycinfo).ab. or (cinahl or cinhal).ab. or science citation index.ab. or bids.ab. or cancerlit.ab. or reference list$.ab. or bibliograph$.ab. or hand-search$.ab. or relevant journals.ab. or manual search$.ab. or ((selection criteria or data extraction).ab. and review/)
15. exp clinical pathway/ or exp clinical protocol/ or exp consensus/ or exp consensus development conference/ or exp consensus development conferences as topic/ or critical pathways/ or exp guideline/ or guidelines as topic/ or exp practice guideline/ or practice guidelines as topic/ or health planning guidelines/ or (guideline or practice guideline or consensus development conference or consensus development conference, NIH).pt. or (position statement* or policy statement* or practice parameter* or best practice*).ti,ab,kf,kw. or (standards or guideline or guidelines).ti,kf,kw. or ((practice or treatment* or clinical) adj guideline*).ab. or (CPG or CPGs).ti. or consensus*.ti,kf,kw. or consensus*.ab. /freq=2 or ((critical or clinical or practice) adj2 (path or paths or pathway or pathways or protocol*)).ti,ab,kf,kw. or recommendat*.ti,kf,kw. or (care adj2 (standard or path or paths or pathway or pathways or map or maps or plan or plans)).ti,ab,kf,kw. or (algorithm* adj2 (screening or examination or test or tested or testing or assessment* or diagnosis or diagnoses or diagnosed or diagnosing)).ti,ab,kf,kw. or (algorithm* adj2 (pharmacotherap* or chemotherap* or chemotreatment* or therap* or treatment* or intervention*)).ti,ab,kf,kw.
16. exp comment/ or exp editorial/ or exp "review"/ or case reports/ or meta-analysis/ or RANDOMIZED CONTROLLED TRIAL/ or exp CLINICAL TRIAL/ or exp historical article/ or exp PUBLISHED ERRATUM/
17. exp PRACTICE GUIDELINE/ or exp GUIDELINE/
18. exp Genetics/ or exp POLYMORPHISM, GENETIC/
19. (in vitro or assay$ or biomarker$).ti,ab.
20. exp animals/ not humans.sh.
21. (genetic$ or gene or polymorph$ or allele$).ti.
22. (rat or rats or mice or mouse).ti,ab.
23. (randomized controlled trial or controlled clinical trial).pt. or placebo$.ab. or clinical trials as topic.sh. or random$.ab. or trial$.ti.
24. 13 or 14 or 15 or 16 or 17 or 18 or 19 or 20 or 21 or 22 or 23
25. 12 not 24
26. limit 25 to yr="2008-Current"

Supplementary Table 2: Data extracted from the studies

**General information**	
Type of disease	Rheumatoid arthritis<br>Spondylarthropathies<br>Juvenile idiopathic arthritis<br>Systemic Lupus Erythematosus<br>Osteoarthritis<br>Adult Onset Still Disease<br>Gout<br>Vasculitis<br>Myositis<br>Sjögren Syndrom<br>Other
Number of participants	
Scientific journal ranking in 2018	
Year of publication	
**General information on methods**	
Presence of a method section	Y/N
Description of the data collection	Prospective<br>Retrospective<br>Mixed<br>Not mentioned
Indication of adherence to STROBE guidelines	Y/N
**Exposures and outcomes**	
Treatment was the main exposure	Y/N
Comparison of at least 2 treatment was the main exposure	Y/N
Other outcome than effectiveness (e.g. safety)	Y/N
Type of outcome for effectiveness	Disease activity*<br>Retention<br>Patient reported outcome<br>Radiographic<br>Other
Was the outcome categorical	Y/N
Number of different effectiveness outcome (type and if categorical or not)	
**Confounding**	
Was a crude analysis presented	Y/N
Was an adjusted analysis presented	Y/N
Number of covariates used for adjustment	
Method of selection for adjustment covariates	A priori/wisely<br>Stepwise method<br>Bivariate selection<br>Unknown<br>Other
Method of adjustment for confounding	Multivariate model<br>Stratification<br>Matching<br>Inverse probability weighting

	Propensity score<br>Restriction<br>Other
**Follow-up information and handling of attrition**	
Reporting of patients lost to follow-up	Y/N
Reporting of patients lost to follow-up by treatment	Y/N
Reporting of patients changing/stopping treatment	Y/N
Reporting reasons of treatment discontinuation	Y/N
Handling of attrition in the analysis	Y/N
Method to handle attrition	Non-responder imputation<br>Complete case<br>Last observation carried forward<br>Other
**Missing data**	
Reporting of the number of missing data of covariates of interest	Y/N
Reporting of the method to handle missing data	Y/N
Handling of missing data	Complete case<br>Multiple imputation<br>Last observation carried forward<br>Supplementary category<br>Other
*Disease activity could include a patient reported outcome in the composite score	

Supplementary Table 3: Definitions of some terms used in the review

**A priori/wisely selection of covariates**	Selection of covariates based on background knowledge (e.g. prior studies, causal graph theory, …)
**Attrition**	Type of selection bias that can occur if participants are lost to follow-up or cannot contribute a value of effectiveness
**Bivariate selection**	Selection of covariates in the model based on their association with the outcome in a univariate statistical test. Also called "univariate selection" or "screening"
**Complete case analysis**	Analysis that included only participants with no missing data on the covariates of interests
**Last observation carried forward**	The last observed value of a variables is used for all subsequent observation points if missing
**Loss to follow-up**	Incomplete follow-up, resulting in missing information at the end of the study
**Missing at random**	Missing depending on measured variables but not on unmeasured variables, including missingness in the variable of interest itself
**Non-responder imputation**	Categorisation of subjects no longer under the treatment of interest as non-responders
**Stepwise selection**	Automatic addition or deletion of covariates in the model based on some pre-specified criterion such as a p-value threshold. Stepwise selection can be<br>• Backward: starting with all candidate covariates and deleting them according to criteria<br>• Forward: starting with no variables and adding them according to criteria<br>• A combination of backward elimination and forward selection

Supplementary Table 4. Summary of main results for reporting and analysing for confounding for studies mentioning STROBE vs not mentioning STROBE

	**Studies mentioning STROBE**	**Studies not mentioning STROBE**
N	10	201
**Adjusted and crude analysis presented**	7 (70%)	174 (87%)
**Reporting of patients lost to follow-up**	4 (40%)	65 (32%)
**Reporting of patients changing/stopping treatment**	4 (40%)	9 (48%)
**Handling of attrition in the analysis (in studies with outcome other than retention)**	2/8 (25%)	42/169 (25%)
**Reporting of the number of missing data of covariates of interest**	2 (20%)	34 (17%)
**Reporting of the method to handle missing data**	3 (30%)	60 (30%)

STROBE: STrengthening the Reporting of OBservational studies in Epidemiology.

Supplementary Figure 1: Proportion of studies with more than one outcome for effectiveness by (A) year of publication (B) order of Scientific Journal Rankings from the lowest ranking (1) to the highest (8)

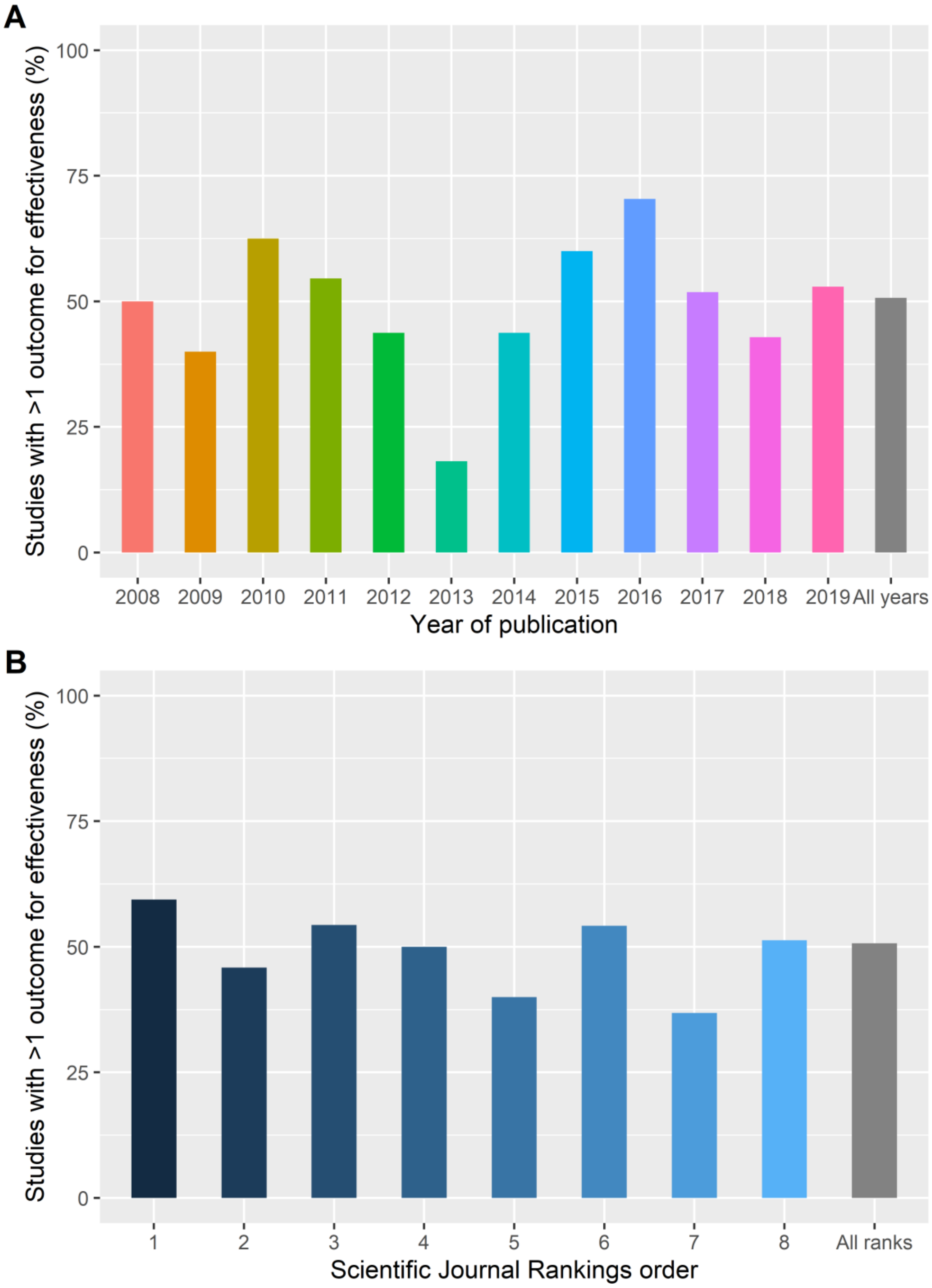

Supplementary Figure 2: Proportion of studies by type of methods for selecting covariates for adjustment and by (A) year of publication (B) order of Scientific Journal Rankings (SJR) from the lowest ranking (1) to the highest (8)

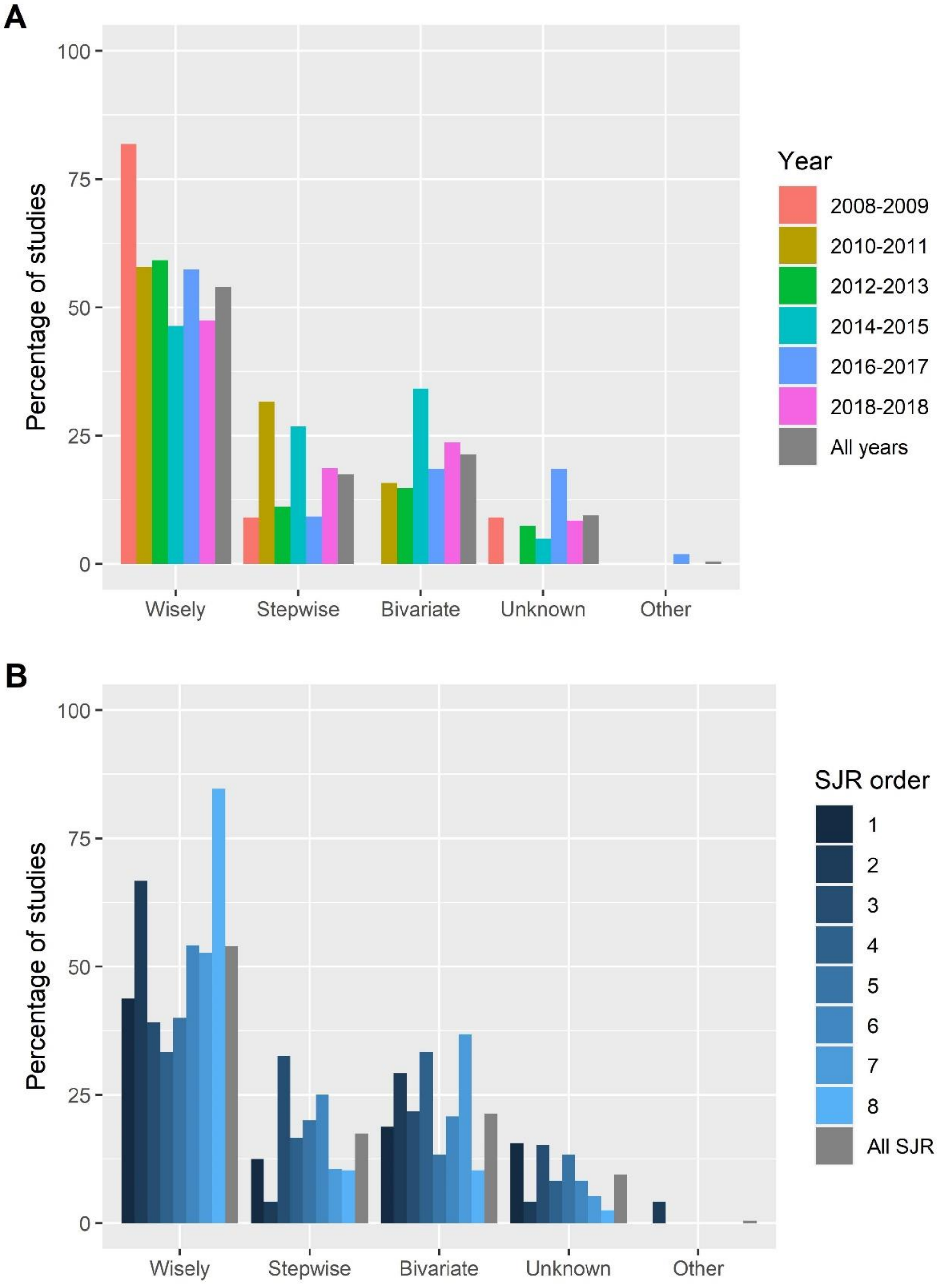

Supplementary Figure 3: Proportion of studies by handling method for and by (A) year of publication (B) order of Scientific Journal Rankings (SJR) from the lowest ranking (1) to the highest (8) in studies with at least 2 treatments as the main exposure and only effectiveness as the outcomes (DS2), with at least one other outcome than retention (n=95)

LOCF: last observation carried forward

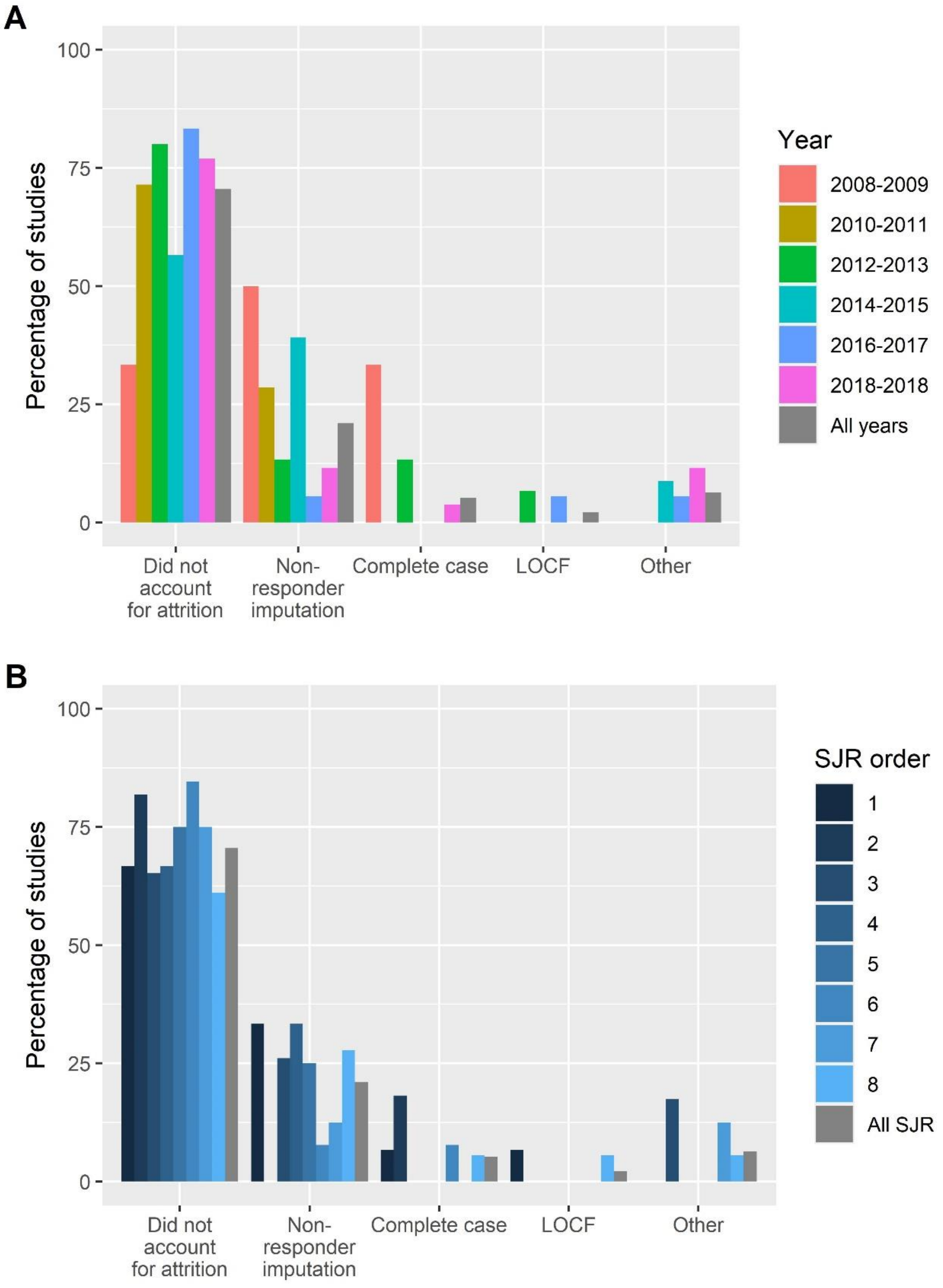

Supplementary Figure 4: Proportion of studies with information on missing data and by type of handling of missing data by (A) year of publication (B) order of Scientific Journal Rankings (SJR) from the lowest ranking (1) to the highest (8) in all studies included (n=211)

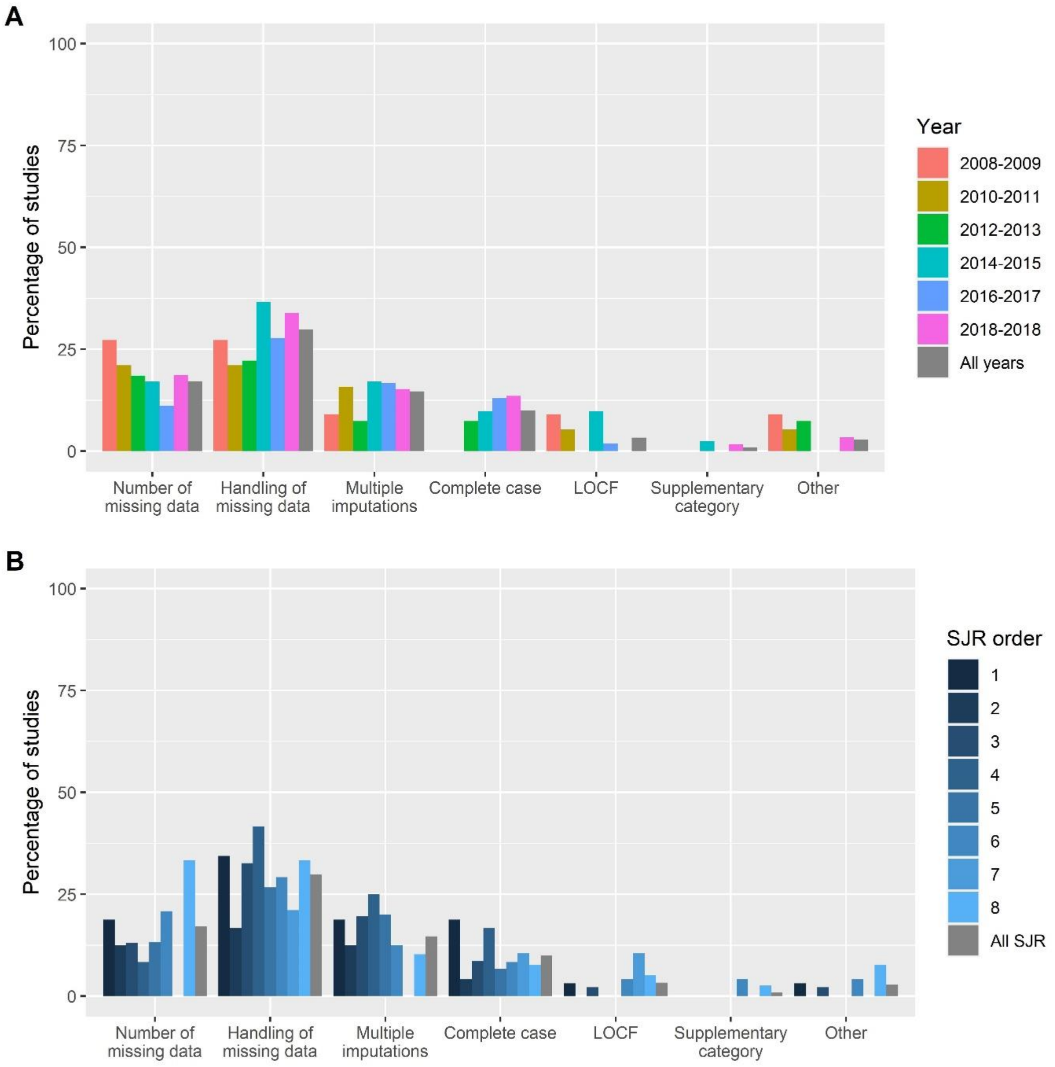

Supplement: Supplementary data [file rmdopen-2021-001818supp001.pdf]
